# Assessing the value-added contributions of community health workers and communities to early child development: a longitudinal study in a low-income setting

**DOI:** 10.1136/bmjph-2024-001192

**Published:** 2025-02-26

**Authors:** Caitlin Hemlock, Maria Dieci, Lia C H Fernald, Emanuela Galasso, Lisy Ratsifandrihamanana, Ann M Weber

**Affiliations:** 1 Department of Environmental and Occupational Health Sciences, University of Washington, Seattle, Washington, USA; 2 Division of Epidemiology, School of Public Health, University of California, Berkeley, Berkeley, California, USA; 3 Department of Health Policy and Management, Emory University, Atlanta, Georgia, USA; 4 Division of Community Health Sciences, School of Public Health, University of California, Berkeley, Berkeley, California, USA; 5 Development Research Group, World Bank, Washington, District of Columbia, USA; 6 Centre Médico-Educatif "Les Orchidées Blanches", Antananarivo, Madagascar; 7 Department of Epidemiology, Biostatistics and Environmental Health, University of Nevada Reno, Reno, Nevada, USA

**Keywords:** Community Health, methods, Epidemiologic Factors

## Abstract

**Background:**

Early child development (ECD) programs in low-resource settings can be effective when delivered through community health workers (CHWs), but there are significant challenges when moving to scale. This analysis aimed to determine the value-added, or relative effectiveness, of CHWs and communities on ECD outcomes within a home-visiting trial and examine associations between observable characteristics of the CHW or community and value-added.

**Methods:**

We analysed data from the four treatment arms of a cluster-randomised trial conducted in 100 communities in rural Madagascar from 2014 to 2016. CHWs (one per cluster) and enrolled children (0–12 months) were surveyed at baseline and 2 years later. Child development scores were assessed using the Ages and Stages Questionnaire-Inventory (ASQ-I) and were internally age-standardised. We determined value-added by estimating CHW/community-level fixed effects on ASQ-I Z-score trajectories (change from baseline to endline), conditional on baseline ASQ-I Z-score and child and household characteristics. We also assessed associations between value-added and observable CHW and community-level characteristics.

**Results:**

We analysed data from 1456 children present at baseline and endline. CHW/community fixed effects explained 26% of ASQ-I trajectory variance and estimates ranged from −1.68 SD to 1.31 SD. CHWs who had another income-generating position were associated with a 0.54 SD (95% CI 0.22, 0.87) increase in ASQ-I Z-score from baseline to endline. Greater increases in children’s ASQ-I Z-scores were also associated with communities that had better healthcare, education and transportation infrastructure and were less geographically dispersed.

**Conclusions:**

Children gained or lost over one standard deviation of ASQ-I Z-score depending on the community and CHW where they lived. Children’s development trajectories benefitted from CHWs involved in an external income-generating activity and communities with better access to healthcare, education, and transportation. Careful consideration of the contexts in which child development interventions are implemented and potential correlates of improved CHW performance are crucial for improved outcomes.

**WHAT IS ALREADY KNOWN ON THIS TOPIC**

- Child development interventions in low-income and middle-income countries are often delivered by community health workers (CHWs), but the influence of CHW and community characteristics on intervention effectiveness remains unclear.

**WHAT THIS STUDY ADDS**

- This study quantifies the causal and joint effect of communities and CHWs on changes in child development over a two-year period in the context of a home-visiting intervention in a low-income country.
- Structural barriers, such as dispersion of the community and access to resources, may impede nutrition and development interventions implemented by CHWs to change child development trajectories.

**HOW THIS STUDY MIGHT AFFECT RESEARCH, PRACTICE OR POLICY**

- Prior to intervention delivery, community-level and CHW-level characteristics that may facilitate or impede the implementation of child development interventions should be assessed. Understanding these factors can help address barriers and optimize pathways to intervention effectiveness, particularly in rural, resource-poor, dispersed settings.

## Background

 Early childhood development (ECD), including language, cognitive and behavioural development, is driven by health, nutrition, responsive caregiving, poverty, the home environment, and many other factors.^1^ In low-income and middle-income countries (LMICs), interventions to improve ECD, such as parenting education or nutritional supplementation, are often delivered by frontline community health workers (CHWs).^2^ CHWs are usually community members without formal medical education but are supported by the health system.^3^ In rural settings where healthcare centres may be far away, CHWs can provide primary healthcare and health education to reduce inequities in access.^4^ CHWs are often employed to implement intervention trials to test scalability and effectiveness in real-world settings.^4^ A review of studies in the USA found that CHWs produced intervention effectiveness, particularly around access to care.^5^ Still, it was noted that research is needed to assess characteristics that make a CHW effective within underserved and hard-to-reach populations. Studies in LMICs have found that specific characteristics can improve CHW performance, including higher education, on-the-job experience, fewer household duties, dependency on income gained from CHW work and how embedded the CHW is in the community.^6 7^ Less is known about how these CHW characteristics impact intervention delivery and downstream health outcomes.

Although there are significant benefits of CHW-delivered ECD programs, these interventions have been difficult to scale.^8^ A previous scaled-up home-visiting trial to improve ECD in rural Madagascar (MAHAY) hypothesised the lack of effectiveness may have been due to household constraints, such as lack of caregiver time to participate in home visit activities or supplies to implement the messaging delivered around play (toys/books).^9^ However, community-level factors, such as spread of the population and geography, may have also constrained the CHWs in their delivery of home visits. Community-level factors could have also played a significant role in ECD, as shown by the high clustering of ECD outcomes by community in the trial results;^9^ investigating these community-related factors can provide additional evidence for pathways to scaling ECD interventions in dispersed rural communities.

Methods in the education and clinical medicine fields leverage a technique called value-added modelling (VAM) to determine the contributions of individual workers in comparison to an average effect.^10^ Rigorous methods are used to understand the relative effectiveness of teachers in improving children’s schooling outcomes,^11^ and nurses have been the subject of VAM to examine changes in patient condition.^12^ In this manuscript, we applied VAM methods to examine the contributions of CHWs in improving child outcomes, focusing on ECD in the context of an intervention that delivered nutritional education, nutritional supplementation and/or early stimulation through CHW home visits. The advantage of VAM is that we can estimate the causal effects of CHWs and the communities they live in on children’s development trajectories by accounting for both observable and unobservable factors at the CHW/community-level over and above child-level factors. From this, we can determine the range of effects that CHWs and communities have on ECD trajectories and the potential impact of improving the value-added of CHW. We can also examine associations between these estimated effects and CHW characteristics to generate hypotheses about intervening pathways to achieving ECD outcomes. In the nursing literature, characteristics associated with better patient outcomes are nurses’ education and experience;^12^ identifying characteristics associated with CHW value-added may be able to separate high and low-performing CHWs in terms of ECD trajectories. This could provide robust evidence to policymakers about the importance of considering CHW-level and community-level factors when implementing community-based programmes.

The objectives of this study were to (1) estimate the value-added, or relative effectiveness, of CHWs and the communities they serve on child development scores among children exposed to a home visiting intervention; and (2) characterise associations between observable CHW and community-level characteristics and estimated value-added, defined as gains in development scores over time.

## Methods

### Study design

We conducted a secondary analysis using data from a multiarm, cluster-randomised controlled trial (cRCT) in rural Madagascar, conducted in 2014–2016,^9 13^ where 125 sites were randomly assigned to 4 treatment arms plus a control arm (1:1:1:1:1 allocation ratio). For the purposes of this observational analysis, we used data from the treatment arms only (100 sites), as our target population was children receiving home visits. Sites were defined as communities where one CHW was already implementing a government-sponsored, community-based health and nutrition programme. An additional CHW was hired for the cRCT to deliver home visits in treatment arms; these added-CHWs were required to live within the community and have completed at least lower secondary education (9 years of formal schooling). We used a value-added approach to understand the effects of added-CHWs on changes in child development outcomes over time, conditional on other community-related factors given the 1:1 allocation of added-CHWs to communities.

### Intervention

The government-sponsored, community-based health and nutrition programme was considered the status quo, and different nutrition and development activities were layered onto home visits in each treatment arm. In the first treatment arm (T1), 14 home visits for intensive nutrition counselling were delivered to pregnant women and children aged 0–24 months. In the second treatment arm (T2), weekly distribution of 20 g sachets of lipid-based nutrient supplementation (LNS) for daily consumption for children 6–18 months was added to the T1 home visits. In the third treatment arm (T3), 40 g sachets were given to all pregnant and lactating women weekly up to 6 months postpartum in addition to 20 g for children 6–18 months. The fourth treatment arm (T4) did not include LNS but added a structured, ECD-focused early stimulation component with fortnightly home visits for children 6–30 months old, adapted from the Reach Up and Learn curriculum for the Malagasy context.^14^ More details on the interventions are included in the published results of the main trial paper.^9^

Added-CHWs received a 10-day training before intervention delivery, which covered listening and communication skills, problem-solving for exclusive breastfeeding, introduction of complementary feeding and food security. They also received periodic refresher training. Added-CHWs in the T4 arm received training on early stimulation and were provided with a week of on-site support by a team of ECD coaches at the beginning of the programme and 6 months thereafter.

### Target population

The study recruited 750 children per arm (30 children per community) starting in the second or third trimester in utero (−6 months) to <12 months in three cohorts: cohort 1 (10 children aged −6 to <0 months), cohort 2 (10 children aged 0 to <6 months) and Cohort 3 (10 children aged 6 to 12 months). For this analysis, we only used children alive at baseline (second or third cohort) with a measure of their developmental status (using the Ages and Stages Questionnaire-Inventory, ASQ-I) in the treatment arms (ie, receiving a home visit). Target children were 24–36 months during the endline assessment (2 years after implementation). Any children who moved during the study period and returned before endline measurement were interviewed and assessed for the main trial but were not included in this analysis because their exposure to the programme was unknown.

### Data collection

Surveys were administered at three time points: baseline, midline (1 year later) and endline (2 years later) (online supplemental figure S1). The household questionnaire, administered to the household head, included information about household demographics and dwelling characteristics.^9^ A questionnaire on prenatal care, nutrition and hygiene knowledge, and the home environment (adapted from UNICEF’s Family Care Indicators [FCI]^15^) was administered to the primary caregiver. Community surveys were administered to key informants for information on population demographics, community resources, and economic and weather-related shocks. The CHW survey included questions on demographics, vocabulary, knowledge, motivation, and employment (described below).

### Added-CHW characteristics

Our characteristics of interest were baseline measurements of added-CHW. Given the previously shown efficacy of the ECD curriculum,^16^ we hypothesized that the value-added of the CHW was driven by the number and quality of home visits delivered, leading to greater intervention impact. We generated a list of variables that could be related to enabling or constraining CHWs, and included CHW education (dichotomised to above secondary II [9 years formal education] and below), age, household wealth, and a measure of receptive vocabulary, measured by the Peabody Picture Vocabulary Test.^17^ We assessed motivation for working as a CHW using a set of 13 items that were scored on a 4-point Likert scale (‘not at all’ to ‘exactly’ the reason) (online supplemental table S1). This 13-item scale was based on a prosocial identity scale and adapted extensively for the context, ease of comprehension and applicability to CHWs.^18^ In a principal components analysis (PCA), all but one item moved in the same direction; thus, we summed the ordinal scores as a measure of the amount of motivation to work as a CHW. In regression analyses, we standardised the sum score so the coefficient would represent a 1 SD increase. Whether the CHW was motivated by compensation moved in the opposite direction as the rest and thus was excluded from the sum score but included as a separate indicator. Finally, we included whether the CHW had another income-generating position since this could reduce the time available for home visits and thus service delivery.

Some communities hired the added-CHW for intervention delivery shortly after baseline survey administration (online supplemental figure S1); for these clusters, we assumed the values reported at midline for the same questions represented baseline values (except for age, which was imputed by subtracting 1 year). We kept clusters where added-CHWs were replaced and included an indicator for turnover in the cluster at any point during the 2-year study period to account for potential breaks in service delivery.

### Community characteristics

Because of the 1:1 allocation of added-CHWs to communities, we examined community location, size, resources, shocks and wealth. Location indicators were rural (yes/no), ‘Hauts Plateaux’ regions (centre highlands, the main agricultural area, versus coastal regions, at greater risk of climate-related shocks) and distance to the regional capital city (kilometres). Population size was ascertained from key informant surveys at baseline and endline and averaged, and a variable for community dispersion was created by summing the distance (kilometres) from all study households to the centre of the community after removing outliers (defined as 1.5 IQR above the 75% percentile). For wealth, we averaged household wealth indices in the community. For resources, we included an indicator for the presence of any market in the community and the average duration of the lean season (period between planting and harvesting seasons with high food and income insecurity). We also generated indices from a PCA of 23 indicators of access to different infrastructure and services in the community (online supplemental table S2), including health and nutrition centres, schools, roads and agricultural resources. We retained components that explained a meaningful amount of variance using the scree plot approach.^19^ For shocks, we included an indicator that the community experienced late rains for the 3 years preceding endline measurement.

### Outcome

The primary outcome of interest was change in ASQ-I scores of enrolled children from baseline to endline among those who received home visits.^20^ The ASQ-I is a caregiver-reported assessment of communication, gross motor, fine motor, personal-social and problem-solving domains. At baseline, 17 items were administered first as caregiver report, then directly observed, resulting in 2 responses per item, combined into a single response (2=yes, can/does do it, 1=sometimes/caregiver says yes, but does not demonstrate, 0=not yet/does not succeed). At endline, items were administered once, but the child was allowed to demonstrate certain skills. The endline coding reflected both caregiver report and observed behaviour to match baseline scoring. Scores were converted to internally age-standardised Z-scores by regressing the ASQ score on age and age-squared and standardising the residuals. We used a complete case analysis approach for missing child outcomes at both time points.

### Child and household covariates

Child-level and household-level covariates included in the covariate-adjusted value-added estimation technique were: household wealth (PCA of asset variables), child age, child sex, maternal education (none, primary, secondary I (less than 9 years of formal schooling), secondary II (at least 9 years of formal schooling), postsecondary), maternal knowledge score (PCA of six continuous measures of maternal knowledge developed to quantify knowledge retention from nutrition education sessions, group activities and/or home visits), household distance to community centre (using global positioning coordinates) and a home environment score constructed using PCA on FCI indicators for engagement in stimulating activities such as story-telling or song-singing.

### Statistical analysis

The original trial calculated a sample size of 25 sites per intervention arm and 30 households, using an ICC of 0.1, alpha of 0.05 and 80%, in order to detect an effect size of 0.3 SD.^9^

Using a value-added estimation technique,^10 21^ we performed ordinary least squares regression on the change in child ASQ-I Z-scores from baseline to endline and included added-CHW/community fixed effects, conditional on the child’s baseline ASQ-I Z-score and child and household covariates (equation 3.1).


(3.1) $$\Delta Y_{ijt}=\beta_{0}Y_{ij0}+\beta_{1}X_{ij0}+E_{j}+\epsilon_{ijt}$$

$\Delta Y_{ijt}$ is the change in ASQ-I Z-score for individual i in community j for time t, $\beta_{0}$ is the coefficient on the baseline ASQ-I Z-score, $X_{ij0}$ is a vector of characteristics for individuals at baseline, $E_{j}$ is a fixed effect at the added-CHW/community-level *j,* and $\epsilon_{ijt}$ is the error term. We adjusted for correlated error structure using robust SEs clustered at the community level. Here, the added-CHW/community fixed effects or value-added are estimated in units of change in ASQ-I Z-score. The joint contribution of added-CHW/community value-added was examined using the change in R^2^ in the regression with and without fixed effects. As a sensitivity analysis, we tested dropping clusters with turnover.

Added-CHW/community fixed effects are estimated relative to one that is arbitrarily selected as a reference category, with potential bias induced from this arbitrary selection. To reduce this potential bias we centred the fixed effects while preserving their ranking and scale.^21 22^ We examined the transformed added-CHW/community fixed effects using descriptive statistics, including range, variance, skewness and kurtosis and tested if they were normally distributed using the Shapiro-Wilk test of normality.^23^

We performed a check of random assignment, a key identifying assumption of the value-added estimation technique.^11^ We tested whether ASQ-I Z-score at baseline was associated with estimated added-CHW/community fixed effects, conditional on child and household characteristics at baseline, using OLS with cluster robust SEs. A coefficient that is not significant suggests that children were randomly sorted into clusters, as measured by child development.

To examine characteristics associated with value-added, we performed a series of three regressions of the estimated and transformed added-CHW/community fixed effects (one observation per community). In the first regression, we included added-CHW characteristics hypothesised to contribute to added-CHW service delivery. Second, we added community-level variables hypothesised to contribute to child development and potentially confound the added-CHW characteristics and child development association. We selected community-level variables by performing a penalised regression of community characteristics on change in ASQ-I Z-score^24^. We used elastic net (setting alpha to 0 for LASSO) and standardised all variables, retaining only those with non-zero coefficients at the lambda value that minimised the mean squared error (determined by cross-validation). Third, we interacted the most important added-CHW variable (determined by the t-statistic in the first model) with treatment arm, adjusting for community characteristics. All regression models used a complete case analysis approach to handle missing data.

Given the design of the additional intervention activities in the T2–T4 arms compared with the T1 arm (additional home visits for LNS distribution (T2+T3) and ECD activities (T4)), we checked if any differences by treatment arm were driven by dispersion of the community by interacting dispersion of the community with treatment arm and the most important added-CHW variable.

PCAs and LASSO were performed in R V.4.2.1 and fixed effects regressions were performed in Stata V.14.

### Patient and public involvement

The home-visiting intervention in the original trial was extensively piloted prior to implementation, including seeking feedback from CHWs and communities. For this secondary analysis, no additional data were collected, thus members of the public were not involved in its development.

## Results

### Sample characteristics

Among the 3738 children in households in 125 communities enrolled in the MAHAY trial at baseline, we used 100 communities (including 100 added-CHWs and 2991 households) from the T1–T4 intervention arms. Among the 2991 enrolled households, 1993 were present at baseline, of which 1928 (97%) had available ASQ measurements. Between baseline and endline, 59 (3%) children died, 255 (13%) were lost to follow-up after baseline and 145 (8%) were lost to follow-up after midline. Among the children available for assessment at endline, 1456 (99%) had available ASQ-I Z-scores.

Demographic characteristics of children were balanced across treatment arms, except for household wealth, where households were less wealthy in T3 (online supplemental table S3). One-quarter of households had mothers with at least secondary I education and the median distance from the child’s household to the community centre was 0.6 km. Children were 6 months of age, on average, at baseline. Added-CHWs (n=100) had a median age of 28 years and 30% had at least secondary I education (table 1). Half had always lived in the community and 78% had another income-generating activity. Fourteen percent of clusters experienced added-CHW turnover. Most added-CHW variables were only weakly correlated with each other (online supplemental figure S2).

**Table 1:** Community health worker (CHW) and community characteristics

	n=100
**CHW characteristics**	
Age (years)	28 (23, 37)
Has 9 years of formal schooling (completed lower secondary)	30 (30%)
Wealth quintile	
Quintile 1	27 (27%)
Quintile 2	15 (15%)
Quintile 3	17 (17%)
Quintile 4	20 (20%)
Quintile 5	20 (20%)
Missing	1
Has always lived in community	50 (50%)
Vocabulary score†	35 (30, 39)
Missing	1
Has another income-generating activity	78 (78%)
Motivation score	37 (34, 43)
CHW turnover	14 (14%)
**Community characteristics**	
Rural	93 (93%)
Located in Hauts Plateaux region of Madagascar	20 (20%)
Distance to regional capital city (km)	30 (13, 50)
Population (per 100, averaged over study period)	13 (8, 17)
Total distance from community centre to study households (km)	24 (15, 34)
Duration of lean season (months, averaged over study period)	5.33 (4.33, 6.00)
Share of communities with 3 years* late rain	22 (22%)
Community-average wealth quintile	3.02 (2.24, 3.78)
Any market at community	28 (28%)

Statistics presented: median (IQR); n (%).

* Three years includes study period and year preceeding.

† Measured by Peabody Picture Vocabulary Test.

### Construction of community access indices

**Figure 1:**
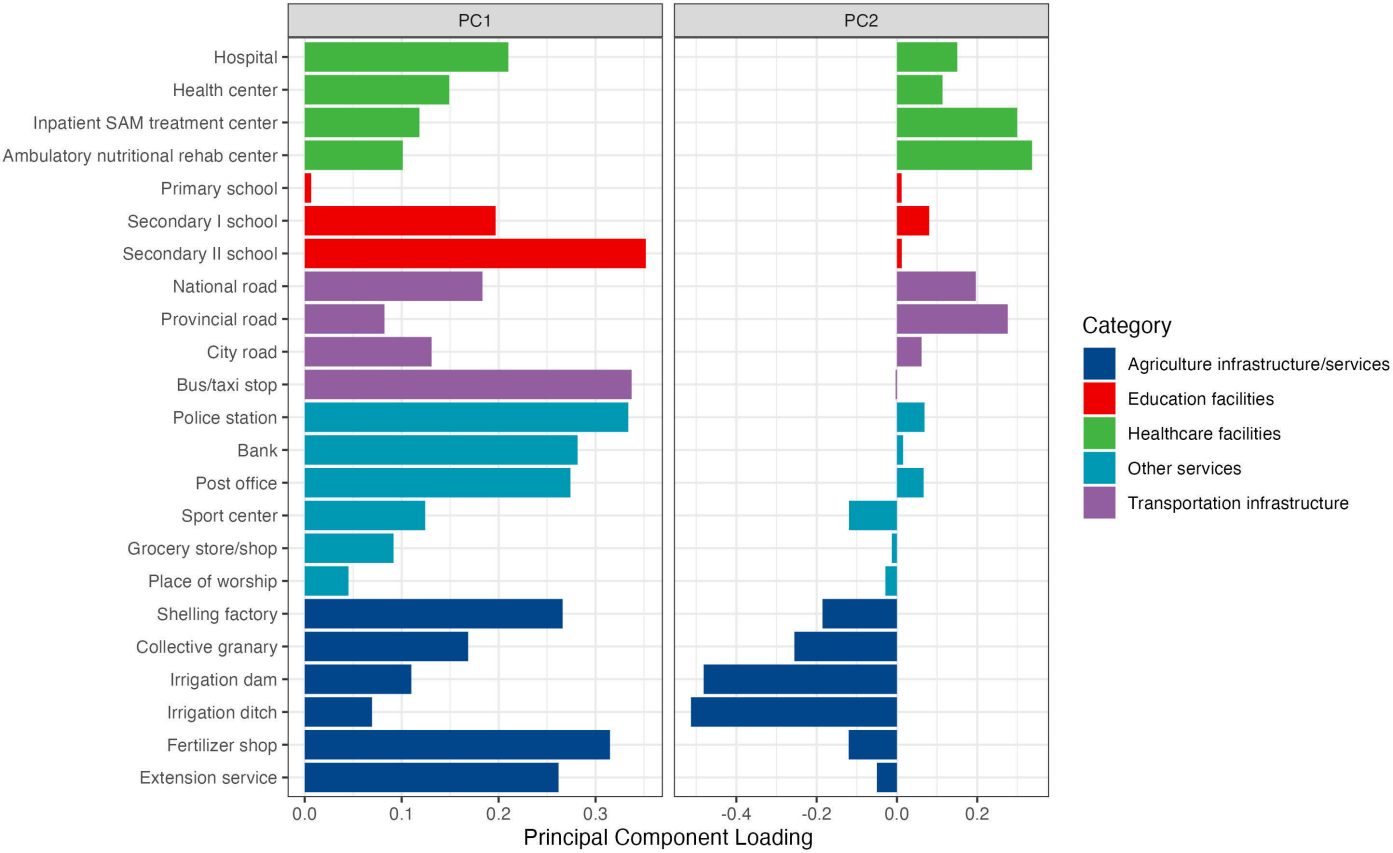
Principal component analysis loadings for community access scores. Principal component analysis was performed on all communities in original trial, including control arm (n=125).

The first two components of the community access PCA explained 49% of the variance (33% and 16%, respectively), with the variance explained levelling off for the remaining 22 components. All variables loaded on the first component (figure 1), which we considered an index of general availability of community resources, with scores ranging from −2.67 to 1.5. The second component separated communities with health, education and transportation infrastructure, assigned positive scores, from communities with more agricultural resources, assigned negative scores, with scores ranging from −1.26 to 1.52.

### Estimating added-CHW/community value-added

The added-CHW/community fixed effects or value-added (referred to hereafter as CHW-CVA) explained 26% of the variance of children’s change in ASQ-I Z-score (online supplemental table S4). The results did not change in our sensitivity analysis of dropping clusters with added-CHW turnover.

**Figure 2:**
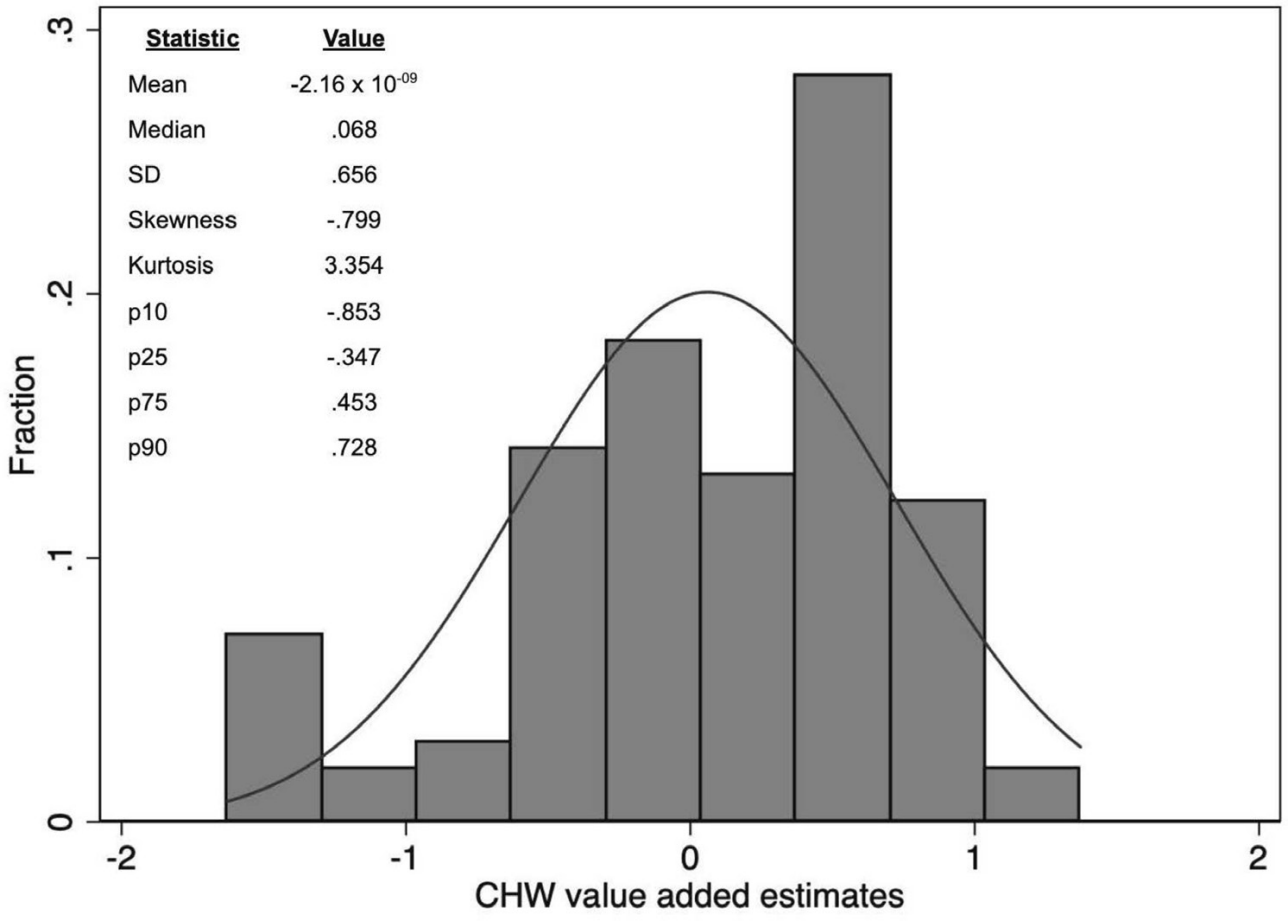
Frequency distribution of CHW/village value-added, n=99. Histogram displays CHW/village fixed effect estimates (VA) with a superimposed normal distribution. VA was estimated by regressing ASQ gain score on child and household-level characteristics with CHW/village fixed effects. ASQ, Ages and Stages Questionnaire; CHW, community health worker.

CHW-CVA coefficients ranged from −1.68 to 1.31 with mean 0 (due to centering transformation) and 0.66 SD. They were distributed non-normally (p<0.01), with negative skewness (−0.8), indicating left-sided tail effects and positive kurtosis (3.33), indicating that the distribution tails are heavier than a normal distribution (figure 2). Added-CHW/communities that ranked in the bottom 20% had a mean change in ASQ-I Z-score of −1.0 SD (SD: 0.48) and those in the top 20% had a mean change in ASQ-I Z-score of 0.77 SD (SD: 0.19). CHW-CVA was not associated with baseline ASQ-I Z-score, indicating the random assignment assumption was met (online supplemental table S5).

### Selection of village characteristics

Community-level characteristics considered influential for change in ASQ-I were both community access indices, population size, distance to the regional capital city, dispersion, three years of late rains, rural location, and Hauts Plateaux region (online supplemental figure S3). The community access index (distinguishing communities with health centres, schools and road infrastructure from agricultural communities) had the highest influence on change in ASQ-I Z-score, with a positive association. The second most influential variable was the community index of overall access to resources, which also had a positive association.

### Characteristics associated with value-added

CHW-CVA was positively associated with whether the added-CHW had another income-generating position and negatively associated with motivation score (table 2, column 1). Added-CHWs with another income-generating position were associated with 0.54 SD increase in change in ASQ-I Z-score (95% CI 0.22, 0.87). The summary motivation score was associated with a −0.17 SD decrease in change in ASQ-I Z-score (per 1 SD increase; 95% CI −0.3, –0.04). Age, always living in the community, education, vocabulary, wealth and turnover were nonsignificant. Added-CHW factors accounted for 22% of the variance in CHW-CVA.

**Table 2:** Results from regressions of the associations between value-added effects and observable community health worker (CHW) and community characteristics

	(1)CHW variables only	(2)Adding community-level variables	(3)Interaction with treatment arm
Treatment arm			
T2	0.16(−0.20, 0.52)	0.10(−0.23, 0.43)	−0.26(−0.98, 0.45)
T3	0.02(−0.34, 0.38)	0.003(−0.33, 0.33)	−0.54(−1.24, 0.16)
T4	0.15(−0.22, 0.52)	0.15(−0.23, 0.52)	−0.44(−1.3, 0.43)
CHW characteristics			
Has another job	0.54***(0.22, 0.87)	0.45***(0.16, 0.75)	0.01(−0.56, 0.59)
Has another job* T2			0.44(−0.35, 1.23)
Has another job* T3			0.67^∧^(−0.11, 1.46)
Has another job* T4			0.69(−0.22, 1.6)
Age (years)	−0.01(−0.025, 0.005)	−0.01(−0.02, 0.01)	0(−0.02, 0.01)
Has always lived in the community	0.24(−0.25, 0.29)	0.12(−0.14, 0.37)	0.11(−0.14, 0.36)
Has 9 years of formal schooling (completed lower secondary)	−0.20(−0.50, 0.09)	−0.22(−0.48, 0.05)	−0.17(−0.44, 0.1)
Motivation score (SD)	−0.17**(−0.30 to –0.04)	−0.12(−0.25, 0)	−0.14**(−0.27 to –0.01)
Vocabulary score	−0.02(−0.03, 0.004)	−0.01(−0.02, 0.01)	−0.01(−0.03, 0.01)
Wealth	0.03(−0.18, 0.25)	0(−0.2, 0.2)	−0.04(−0.25, 0.17)
Turnover in CHW position	−0.06(−0.45, 0.32)	−0.09(−0.48, 0.29)	−0.07(−0.46, 0.32)
Community characteristics			
Total distance from community centre to study households (per 10 km)		−0.10**(−0.19 to –0.01)	−0.09**(−0.19 to –0.004)
Community access score: PC1		0.15**(0.01, 0.28)	0.11(−0.04, 0.25)
Community access score: PC2		0.27***(0.1, 0.45)	0.3***(0.12, 0.48)
Observations*R^2^	980.22	980.45	980.47

Significance levels: *p<0.1, **p<0.05, ***p<0.001, ∧p<0.2 for interaction effects.

Community characteristics included but not presented in the table were whether the community was rural, located in the Hauts Plateaux region, population per 100 (averaged over study period), distance to regional capital city (km), and late rains in the last three years.

Treatment arm reference category is T1.

* Regressions include CHW/community fixed effects as dependent variable (n = 1 dropped arbitrarily in fixed effects estimation and n = 1 dropped from missing CHW characteristics)

The magnitude of association between having another income-generating position and change in ASQ-I Z-score did not change after controlling for community-level factors in the second regression (table 2, column 2). Both community access indices and dispersion of the community were significantly associated with CHW-CVA, and all community-level factors accounted for an additional 23% of the variance. Similar to the penalised regression results, the community infrastructure-related index was positively associated with change in ASQ-I Z-score, suggesting that communities with better health and transportation infrastructure were associated with larger increases in ASQ from baseline to endline; this effect was stronger than the independent effect of overall access to resources (p<0.001). Dispersion of the community was negatively associated with value-added; for every additional 10 km between the community centre and all study households, ASQ change from baseline to endline decreased by 0.10 SD (95% CI −0.19, –0.01).

**Figure 3:**
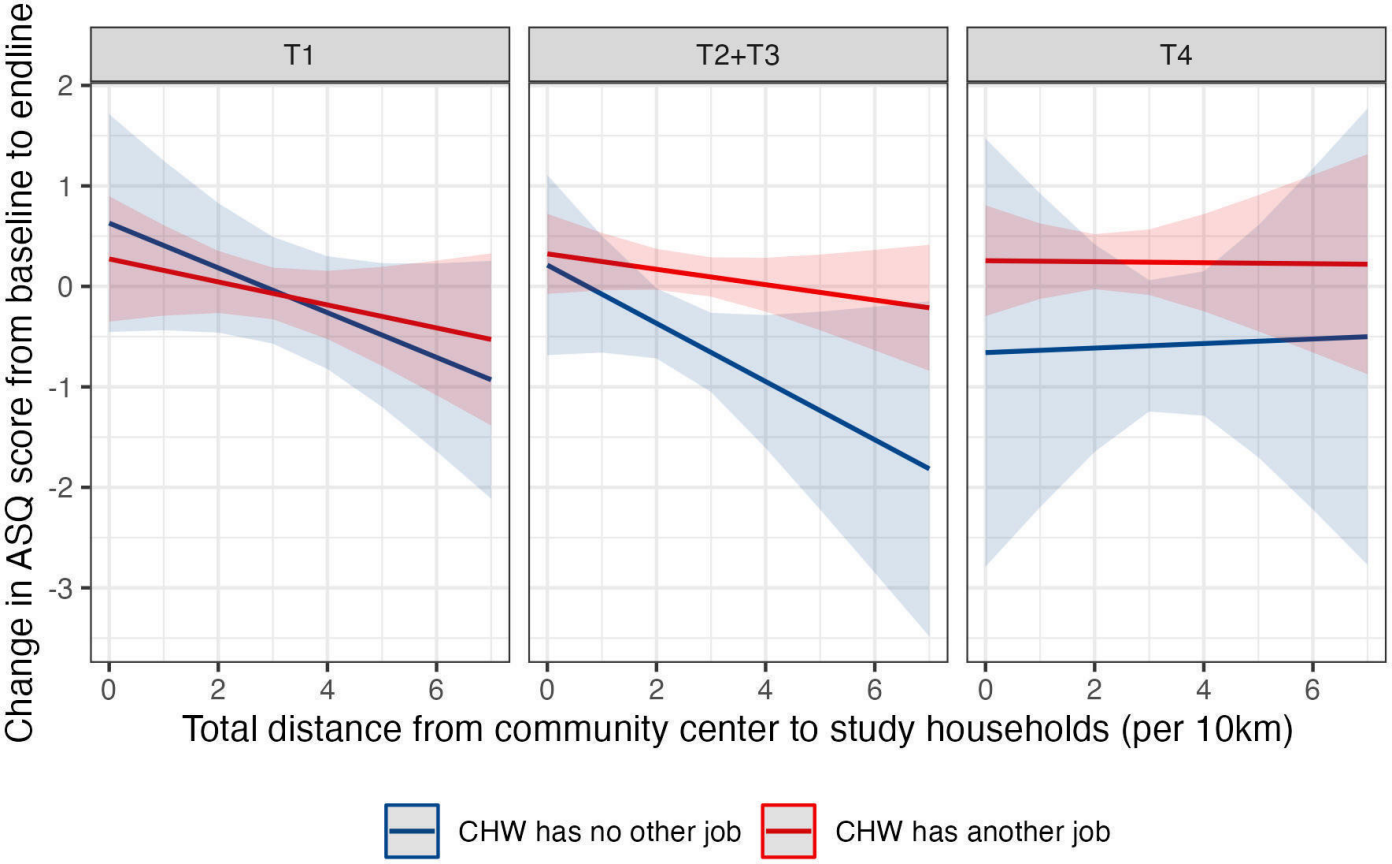
Differences in value-added (CHW/community-level fixed effects) across treatment groups, whether the CHW has another income-generating position, and total distance from community centre to study households. CHW, community health worker.

Finally, in the third regression, we found that value-added was lower in T2, T3 and T4 arms if the added-CHW did not have another income-generating position (table 2, column 3; interaction coefficient only significant for T3 vs T1). We also found that this interaction was modified by the size of the community. As the size of the community increased, the value-added remained positive for added-CHW with another income-generating position, particularly in the LNS (T2+T3) and ECD (T4) interventions, and decreased for added-CHW without another job (figure 3). Compared with added-CHWs with no other job in T1, value-added was 0.17 SD lower per 10 km distance for CHW to travel in LNS communities where the CHW had no other job (95% CI −0.36, 0.01; p=0.06), while value-added was 0.12 SD higher per 10 km distance in ECD communities where the CHW had another job (95% CI −0.05, 0.3; p=0.17).

## Discussion

In summary, we found that CHWs and the communities in which children live have strong effects on child development trajectories in the first 2 years, as shown in the context of rural Madagascar within a home visiting intervention. CHW/community value-added effects were wide-ranging, with children gaining or losing over 1 SD of additional ASQ-I Z-score over the 2-year period depending on the characteristics of the community and added-CHWs. Added-CHWs having another job was associated with an additional 0.45 SD increase in ASQ-I Z-score over 2 years, and this finding persisted after controlling for community-level factors that may be related to job opportunities in the community. We also found that community infrastructure such as healthcare, schools and major roads was positively associated with value-added and was the most important variable across a range of community factors related to location, size, shocks and access to community resources. A negative association between site dispersion and change in ASQ-I Z-score was attenuated in treatment groups with LNS supplementation or ECD stimulation, but only where added-CHWs had another income-generating position.

The only CHW characteristic positively associated with value-added was whether the CHW had another income-generating position, and the magnitude of this effect on ASQ-I Z-score was similar to programmes that have delivered the Reach Up programme through home visits.^16^ We hypothesised that having another position for generating income would result in lower time to dedicate to the CHW workload, as found in India.^25^ However, having another income-generating position could be related to other dimensions that affect the quality of the caregiver interactions and willingness of the caregiver to implement messages from the programme. For example, CHWs having another income-generating position could be a proxy for CHW status in the community and subsequent respect for the messages being delivered. Several studies found that social ‘prestige’ or the level of recognition CHWs received from their CHW work was a strong motivator.^26^,^28^ In multiple countries in Africa and Asia, CHWs reported that being perceived as knowledgeable and receiving social recognition were particularly important for motivation,^28^ while in Dhaka urban slums, social prestige was associated with performing CHW activities, including home visits, more frequently.^26^ Prestige may be a better proxy for motivation as it relates to attitude or personality about work, compared with our construction of motivation. As noted above, having another job buffered the negative effects of dispersion of the community on ASQ-I Z-scores, which may be tied into the motivation or willingness to travel far distances for high-frequency home visits. Having another income-generating position may be related to CHWs interest in career advancement, which was found to be beneficial for both service delivery and health outcomes in an RCT in Zambia,^29^ or reduction of economic stress, giving the ability to invest their own financial resources in CHW activities. Lastly, the majority of the non-CHW income-generating positions were related to agriculture (data not presented), which could mean that other income-generating agriculture work was conducted early in the mornings with time for CHW activities during the day. Future work from our group, including a cRCT that measures CHW time use, will study how CHWs use their time and how other income-generating positions and time constraints may play a part in the implementation of ECD interventions.^30^

Part of the original hypothesis for the lack of effectiveness of the scaled-up home intervention in Madagascar^9^ was supply-side constraints, including long distances for CHW to travel for weekly or biweekly visits, impeding the fidelity of the intervention. Our empirical findings corroborate the notion that the dispersion of the community (ie, distance to all households in the study from the centre of the community) was significantly associated with a decrease in ASQ-I Z-score from baseline to endline. A recent systematic review of perceived workloads by CHWs found that lack of transport to perform home visits, especially in areas with long distances to walk due to remoteness of households, contributed to having a perception of a high workload.^31^ Similarly, a qualitative study of different CHW cadres found that CHWs in dispersed and hilly regions of India, as in our study areas, complained of excessive workloads due to transportation challenges.^28^ The negative effects of dispersion could also be related to demand-side constraints. One qualitative study in Bangladesh noted that households that were farther away were less familiar with the CHW and thus potentially less willing to receive messages, and that CHWs did not visit households of certain religions.^32^ Similarly, another study in Bangladesh noted households closer to the CHW had closer interpersonal relationships and interacted on a more informal basis.^33^ An important dimension not measured in our study was the quality of interactions and caregivers’ perceptions of the CHW and openness to receive messages about nutrition and ECD.^34^ We found some of these distance-related effects were buffered among children in the treatment arms where LNS and ECD stimulation were delivered, but only where CHWs had another income-generating position, potentially suggesting that supplementation or stimulating activities delivered by high-performing CHW can mitigate the effects of remoteness.

An alternative explanation for the interaction effects we found between community dispersion and the CHW having another job could be that communities in which CHWs have another job are better off socioeconomically, which in turn could promote child development. Headey *et al* found that the harmful effects of remoteness on linear physical growth in 23 sub-Saharan countries were mediated by parental education, wealth and infrastructure services.^35^ We found that communities with health, education and transportation infrastructure were positively associated with improvements in child development compared with more agriculturally focused communities, and that differentiation was the most important community variable among all examined. The existence of health, education and transport infrastructure has downstream effects on development projects; their presence familiarises the community with the use of services, which not only promotes access but encourages willingness to overcome mistrust and take up new services,^36^ such as ECD stimulation. As a result, caregivers and CHWs may be more engaged in activities and better able to assimilate the messages transmitted, resulting in a higher impact of the intervention.

Our study had several limitations. First, while we controlled for many factors related to community socioeconomic status, it may be that our association between CHWs having another income-generating position and improved child development is confounded at the community level, particularly by constructs that we were unable to assess or control for in the analyses. We could not identify CHW effects separately from community effects since CHWs are hired from the community population and do not have determined exposure periods to children (such as teachers do for one school year). However, a benefit to using VAM is that we can isolate the effects of the environment after adjusting for individual-level factors and other random variation.^10^ Thus, we find that the association with added-CHW/community-level factors is present after controlling for household-level factors typically associated with child development such as maternal education and knowledge, wealth and the home environment.^1^ Since this was a secondary data analysis, it may be that the variables chosen and the way they were categorised were imperfect proxies of the constructs of interest, for example, with CHW motivation. Additionally, we did not assess mechanisms for these results, including associations between value-added and delivery of home visits. The original trial did not log the number and quality of home visits during the intervention delivery, given the administrative burden of this task on CHWs and supervisors. The trial did ask caregivers to report the length of the most recent visit and the number of visits in the previous month, however, these were only asked once per year (during the midline and endline surveys), and are not necessarily correlated with quality; thus we decided not to assess these variables. We also lacked data for the number of hours CHWs spent travelling to households or for their other income-generating position and thus cannot assess associations between having another income-generating position and time constraints or workload. Future research will elucidate how CHWs use their time and correlations between time constraints and barriers to intervention implementation.^30^

## Conclusions

In conclusion, we recommend careful consideration of the environments in which ECD interventions are implemented and factors that impede or facilitate intervention delivery by CHWs. We found large differences in the value-added of CHW/communities associated with CHWs having another income-generating position, community dispersion and community access to healthcare, education and transportation infrastructure. Examining the settings for implementing ECD interventions is crucial for identifying communities with significant barriers to achieving desired outcomes, given the nearly two SD difference in value-added between the bottom and top 20%and a high proportion of variance in child development growth over two years accounted for by CHW/community fixed effects. Future research, particularly in very rural contexts such as Madagascar, should examine community-level correlates of child development further, as well as explore the potential benefit of community-level interventions.

## Supplementary material

10.1136/bmjph-2024-001192 online supplemental file 1

## Data Availability

Data are available on reasonable request.
