# Supplementary material for: Assessing the value-added contributions of community health workers and communities to early child development: a longitudinal study in a low-income setting
Source: BMJ Public Health. 2025 Feb 26;3(1):e001192. doi: 10.1136/bmjph-2024-001192 (PMC11865798; doi:10.1136/bmjph-2024-001192)
Supplement: online supplemental file 1 [file bmjph-3-1-s001.docx]

Figure S1. Trial and data collection timeline.


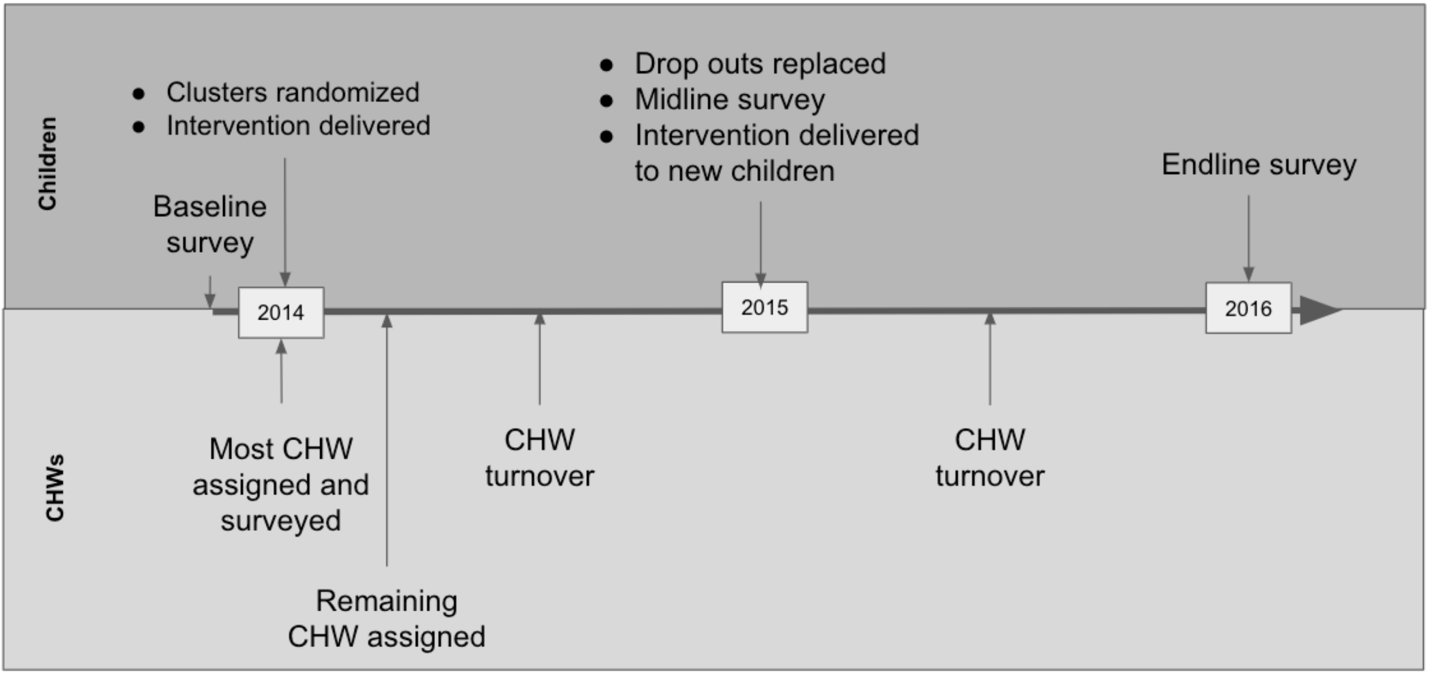


Table S1. Items assessed for motivation score.

| **Item** | **Motivation assessed** |
| --- | --- |
| For the approval I get from others | Extrinsic |
| Because I would feel really bad if I didn’t | Intrinsic |
| Because I feel it is important to me | Intrinsic |
| Because this work corresponds to my personal values | Intrinsic |
| Because I feel considered by my family and by others | Extrinsic |
| Because I would feel guilty otherwise | Intrinsic |
| Because it makes me proud and makes me a worthy person | Extrinsic |
| Because the work of CHW is a fundamental part of my personality | Intrinsic |
| Because I am listened to by my community | Extrinsic |
| Because I enjoy doing my job | Intrinsic |
| Because this work helps me to participate in village development | Extrinsic |
| Because I love my job and I find it interesting | Intrinsic |
| For compensation | Extrinsic |
|  | |

Table S2. Availability of community-level resources and infrastructure, by site.

|  | n = 125 |
| --- | --- |
| **Healthcare facilities** |  |
| Hospital | 18 (14%) |
| Health center | 101 (81%) |
| Inpatient SAM treatment center | 23 (18%) |
| Ambulatory nutritional rehab center | 30 (24%) |
| **Education facilities** |  |
| Primary school | 124 (99%) |
| Secondary I school | 94 (75%) |
| Secondary II school | 43 (34%) |
| **Transportation infrastructure** |  |
| National road | 40 (32%) |
| Provincial road | 32 (26%) |
| City road | 93 (74%) |
| Bus/taxi stop | 41 (33%) |
| **Other services** |  |
| Police station | 38 (30%) |
| Bank | 26 (21%) |
| Post office | 28 (22%) |
| Sport center | 94 (75%) |
| Grocery store/shop | 109 (87%) |
| Place of worship | 119 (95%) |
| **Agriculture infrastructure/services** |  |
| Shelling factory | 40 (32%) |
| Collective granary | 32 (26%) |
| Irrigation dam | 52 (42%) |
| Irrigation ditch | 64 (51%) |
| Fertilizer shop | 33 (26%) |
| Extension service | 29 (33%) |
| Note: statistics presented: n (%). SAM = severe acute malnutrition. | |

Table S3. Baseline characteristics of children and households among children enrolled in the home visiting program.

|  | Overall  n = 1,456 | T1  n = 397 | T2  n = 342 | T3  n = 388 | T4  n = 329 |
| --- | --- | --- | --- | --- | --- |
| Age (months) | 6.0 (3.0, 8.0) | 6.0 (3.0, 8.0) | 6.0 (3.0, 8.8) | 6.0 (3.0, 8.2) | 5.0 (3.0, 8.0) |
| Male child | 733 (50%) | 204 (51%) | 164 (48%) | 192 (49%) | 173 (53%) |
| Maternal education |  |  |  |  |  |
| Did not attend school | 371 (25%) | 116 (29%) | 94 (27%) | 85 (22%) | 76 (23%) |
| Primary or less | 766 (53%) | 192 (48%) | 179 (52%) | 223 (57%) | 172 (52%) |
| Secondary I | 248 (17%) | 68 (17%) | 57 (17%) | 65 (17%) | 58 (18%) |
| Secondary II | 71 (4.9%) | 21 (5.3%) | 12 (3.5%) | 15 (3.9%) | 23 (7.0%) |
| Maternal knowledge score | -0.27 (-0.89, 0.79) | -0.31 (-0.89, 0.79) | -0.29 (-0.89, 0.78) | 0.16 (-0.85, 0.82) | -0.26 (-0.85, 0.84) |
| Home environment score | -0.04 (-0.69, 0.72) | 0.05 (-0.54, 0.71) | 0.08 (-0.67, 0.84) | -0.18 (-0.78, 0.56) | -0.20 (-0.74, 0.72) |
| Missing | 6 | 4 | 1 | 0 | 1 |
| Wealth quintile |  |  |  |  |  |
| 1 | 291 (20%) | 76 (19%) | 60 (18%) | 97 (25%) | 58 (18%) |
| 2 | 290 (20%) | 72 (18%) | 71 (21%) | 87 (23%) | 60 (18%) |
| 3 | 290 (20%) | 68 (17%) | 74 (22%) | 80 (21%) | 68 (21%) |
| 4 | 290 (20%) | 79 (20%) | 78 (23%) | 83 (22%) | 50 (15%) |
| 5 | 291 (20%) | 102 (26%) | 59 (17%) | 37 (9.6%) | 93 (28%) |
| Missing | 4 | 0 | 0 | 4 | 0 |
| Distance from child household to site center (km) | 0.63 (0.16, 1.40) | 0.67 (0.25, 1.50) | 0.40 (0.14, 1.12) | 0.75 (0.17, 1.46) | 0.47 (0.15, 1.25) |
| Baseline ASQ-I Z-score | 0.01 (-0.58, 0.66) | 0.01 (-0.58, 0.66) | 0.13 (-0.51, 0.75) | -0.08 (-0.69, 0.55) | -0.01 (-0.49, 0.68) |
| Note: statistics presented: n (%); Median (IQR) | | | | | |

Figure S2. Correlation between CHW characteristics.


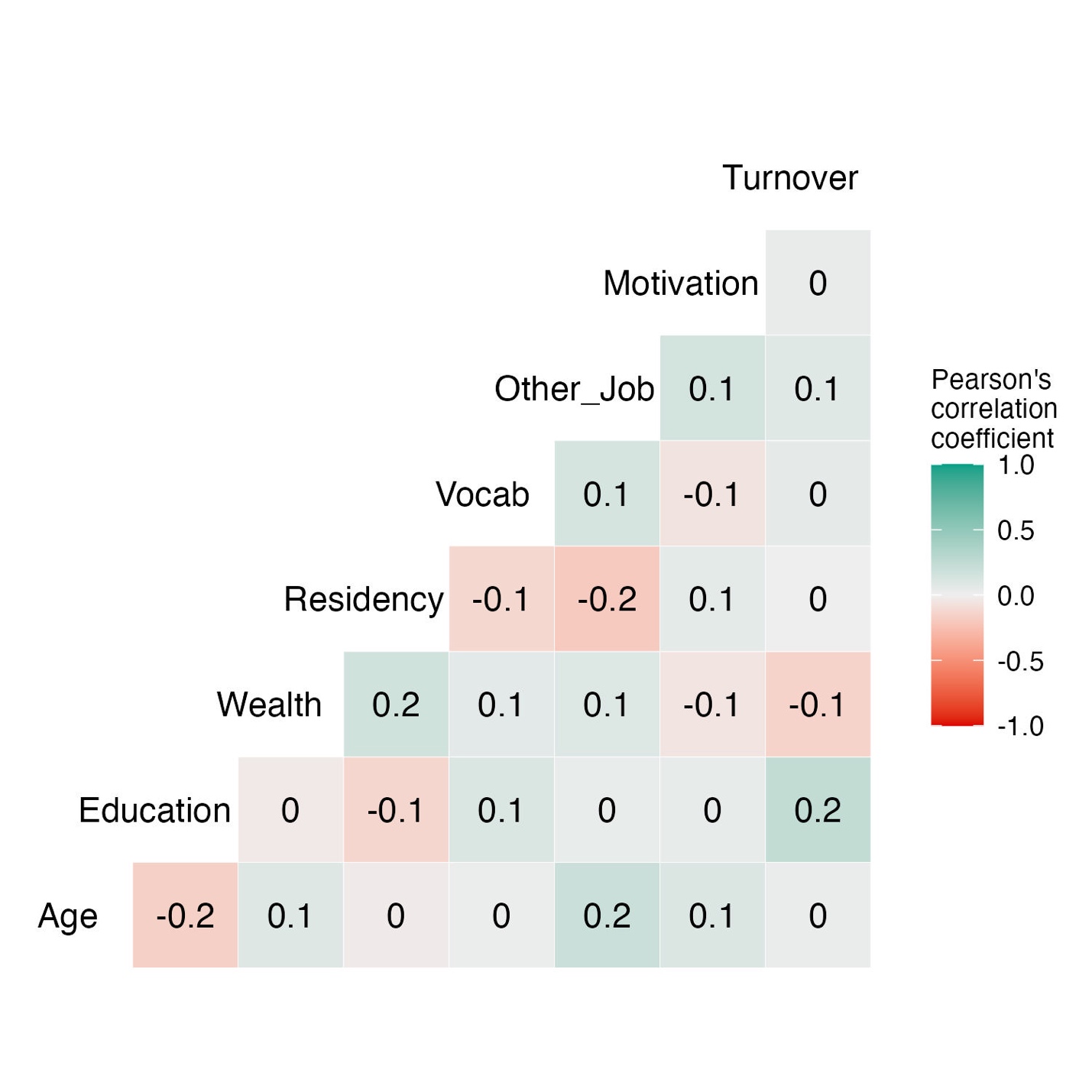


Table S4. Estimating community health worker/community fixed effects using change in ASQ-I Z-score from baseline to endline.

|  | (1) CHW/community fixed effects | (2)  Dropping fixed effects |
| --- | --- | --- |
| Baseline ASQ-I Z-score | -0.91*** (-0.97, -0.85) | -0.89***  (-0.96, -0.82) |
| Age (months) | 0.00  (-0.01, 0.02) | -0.01  (-0.03, 0.01) |
| Child is female | 0.02  (-0.05, 0.09) | 0.04  (-0.05, 0.13) |
| Maternal education |  |  |
| Did not attend school | - | - |
| Primary or less | -0.10*  (-0.02, 0.22) | -0.08  (-0.26, 0.10) |
| Secondary I | 0.31***  (0.16, 0.45) | 0.14  (-0.09, 0.37) |
| Secondary II | 0.39***  (0.21, 0.58) | 0.14  (-0.22, 0.50) |
| Household wealth index | 0.06***  (0.04, 0.09) | 0.02  (-0.00, 0.05) |
| Home environment score | 0.06  (-0.01, 0.14) | 0.18***  (0.07, 0.28) |
| Maternal knowledge score | 0.02  (-0.01, 0.06) | 0.02  (-0.06, 0.09) |
| Distance from child household to site center (km) | 0.01  (-0.02, 0.04) | -0.04  (-0.09, 0.02) |
| Constant | -0.18***  (-0.03, -0.05) | 0.07  (-0.11, 0.24) |
| Observations  R2  Site FE | 1446  0.70  99 | 1446  0.44  0 |
| Significance levels; * p < 0.1, ** p < 0.05, *** p < 0.001 | |  |
|  | |  |

Table S5. Association between value-added and baseline ASQ-I Z-scores to assess value-added estimation assumption of randomization.

|  | Baseline  ASQ-I Z-Score |
| --- | --- |
| CHW/Community value-added | 0.04 (-0.08, 0.16) |
| Treatment Arm |  |
| T1 | - |
| T2 | 0.13 (-0.07, 0.32) |
| T3 | 0.02 (-0.21,0.25) |
| T4 | 0.06 (-0.15, 0.28) |
| Age (months) | -0.04** (-0.06, -0.02) |
| Child is female | 0.07 (-0.02, 0.16) |
| Maternal education |  |
| Did not attend school | - |
| Primary or less | -0.00 (-0.15, 0.14) |
| Secondary I | -0.04 (-0.21, 0.13) |
| Secondary II | -0.03 (-0.32, 0.26) |
| Household wealth index | 0.03 (-0.01, 0.06) |
| Home environment score | 0.38** (0.28, 0.48) |
| Maternal knowledge score | 0.03 (-0.01, 0.08) |
| Distance from child household to site center (km) | 0.02 (-0.02, 0.07) |
| Constant | 0.17 (-0.05, 0.38) |
| Observations  R2 | 1446  0.11 |
| Significance levels; * p < 0.1, ** p < 0.05, *** p < 0.001 | |
|  | |

Figure S3. Results of penalized regression of community-level factors on change in ASQ-I Z-score.


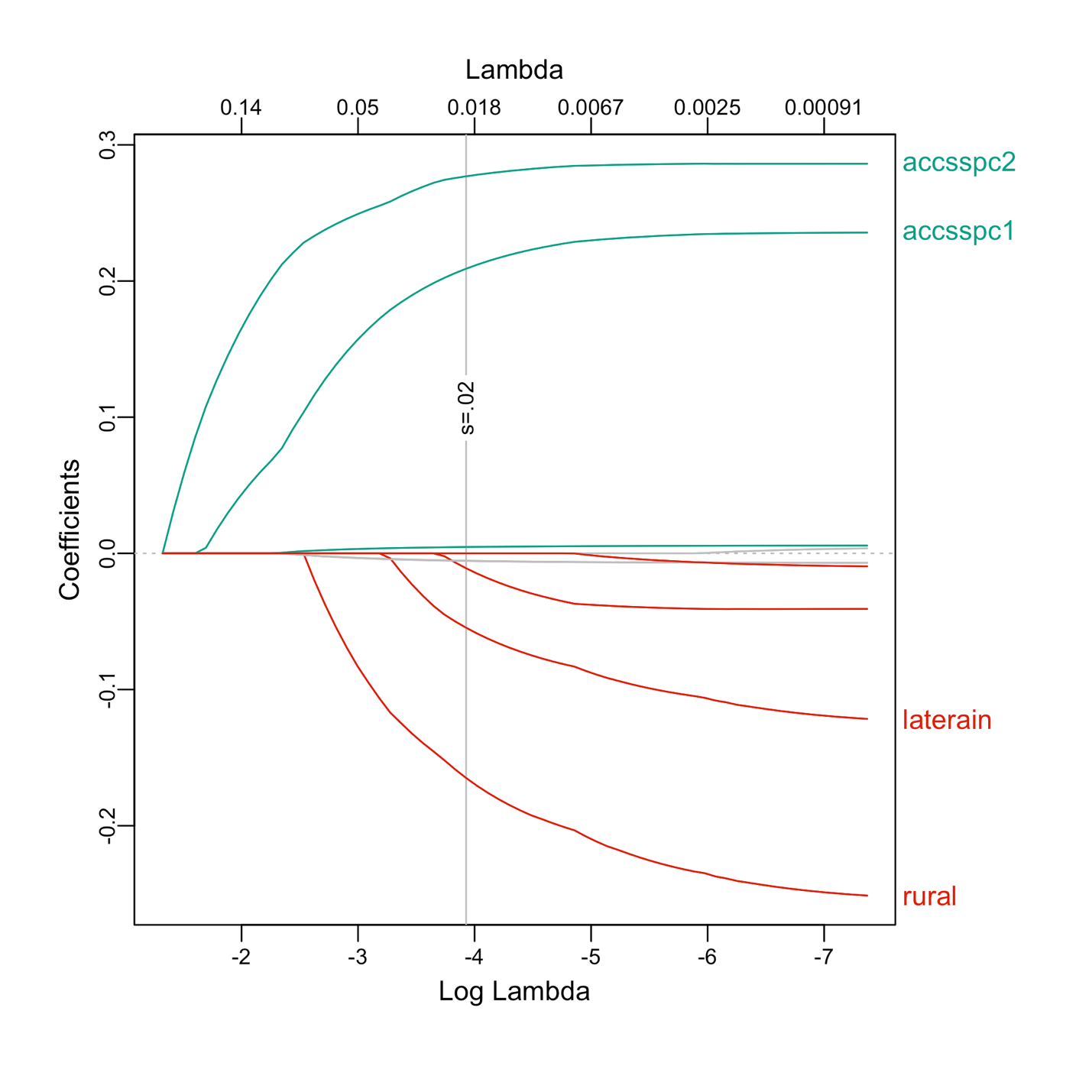


Note: Penalized regression of difference in ASQ-I Z-score from baseline to endline on standardized community-level variables conducted via LASSO. Value for lambda (shrinkage coefficient [s]) was chosen through cross-validation (value that minimizes mean squared error).
